# MKlp2 inhibitior paprotrain affects polar body extrusion during mouse oocyte maturation

**DOI:** 10.1186/1477-7827-11-117

**Published:** 2013-12-21

**Authors:** Jun Liu, Qiao-Chu Wang, Xiang-Shun Cui, Zhen-Bo Wang, Nam-Hyung Kim, Shao-Chen Sun

**Affiliations:** 1 College of Animal Sciences and Technology, Nanjing Agricultural University, 210095 Nanjing, China; 2 Department of Animal Science, Chungbuk National University, 361-763 Cheongju, Korea; 3 State Key Laboratory of Reproductive Biology, Institute of Zoology, Chinese Academy of Sciences, 100101 Beijing, China

**Keywords:** MKlp2, Meiosis, Paprotrain, Polar body

## Abstract

**Background:**

Mammalian oocyte meiotic maturation involves a number of important processes, including spindle assembly and migration, cortical reorganization and polar body extrusion. Numerous proteins contribute to these processes, but it is unknown whether MKlp2 (mitotic kinesin-like protein 2; also called KIF20A), a microtubule-associated protein that regulates cytokinesis during mitosis, is involved in oocyte maturation.

**Methods:**

Confocal microscopy, time lapse microscopy, inhibitor treatment were adopted to examine the roles of MKlp2 in mouse oocyte.

**Results:**

Immunostaining results showed that MKlp2 localized to oocyte microtubules. Time-lapse microscopy showed that disrupting MKlp2 expression with paprotrain, a specific inhibitor of MKlp2, resulted in polar body extrusion failure. This could be rescued after rescuing oocytes from paprotrain in fresh medium. Cell cycle analysis showed that most oocytes were arrested at metaphase I or telophase I. However, oocyte spindle structure and chromosome alignment were not disrupted after the inhibition of MKlp2 by paprotrain.

**Conclusions:**

This study demonstrated that MKlp2 is crucial for oocyte maturation by regulating polar body extrusion.

## Background

During meiosis, accurate chromosome separation ensures the proper distribution of genetic materials [1]. After chromosome separation during mouse oocyte meiotic maturation, the primary oocyte generates two daughter cells, a small polar body and a highly polarized big metaphase II (MII)-arrested oocyte that awaits fertilization. Based on chromosome separation and cytokinesis, small polar body extrusion is essential for the retention of maternal components for early embryo development [2].

Kinesins are a large family of proteins that share a conserved motor domain, which binds to microtubules and couples ATP hydrolysis to mechanical force generation [3]. The kinesin family plays various roles in cellular functions, including mitotic spindle formation and chromosome partitioning, as well as intracellular movements of organelles and vesicles [3,4]. The kinesins MKlp1, MKlp2 and MPP1 (also called KIF23, KIF20A and KIF20B, respectively) are subcategorized into the kinesin-6 family [5]. All three of these members have functions during cytokinesis [6-8].

MKlp2 was first reported to be involved with Golgi apparatus dynamics through its direct interactions with Rab6, a small GTPase [9]. Previous studies showed that MKlp2 localized to the midzone of the spindle during anaphase and to the cleavage furrow and midbody during telophase in mitotic cells [8,10]. MKLP2 is crucial for the relocation of the chromosomal passenger complex (CPC) from the centromere to the central spindle [11-13] and for the appropriate localization of polo-like kinase 1 (PLK1) to the spindle midzone [14,15]. MKlp2 also promotes the microtubule-dependent localization of Cdc14A to the central spindle during anaphase [12]. As a novel binding partner of Mad2, MKlp2 is inhibited to load onto the mitotic spindle by Mad2. Controlling MKlp2 by Mad2 is important for proper mitotic progression and cytokinesis [16].

Although MKlp2 is known to function in mitotic spindle and cytokinesis, its role in oocyte meiotic maturation has not been discovered. In this study, we demonstrate that MKlp2 is involved in mouse oocytes meiotic maturation and disruption of MKlp2 results in the failure of polar body extrusion.

## Methods

### Antibodies and chemicals

Rabbit polyclonal anti-MKlp2 antibody was purchased from Santa Cruz (Santa Cruz, CA). Paprotrain, a cell-permeable acrylonitrile compound that inhibits MKlp2, was purchased from Calbiochem (Merck KGaA, Darmstadt, Germany). Mouse polyclonal anti-α-tubulin-FITC antibody was purchased from Sigma (St Louis, MO). Goat anti-rabbit IgG/FITC and goat anti-rabbit IgG/TRITC were purchased from Zhongshan Golden Bridge Biotechnology Co., Ltd (Beijing).

### Oocyte collection and culture

All animal manipulations were conducted according to the guidelines of the Animal Research Committee of Nanjing Agricultural University. Mice were sacrificed by cervical dislocation. Germinal vesicle-intact oocytes were collected from ovaries of 4- to 6-week-old ICR mice and cultured in M2 medium (Sigma Chemical Co., St. Louis, MO) without cumulus cells under paraffin oil at 37°C in a 5% CO_2_ atmosphere. Oocytes were used for immunostaining after different times in culture.

### Nocodazole treatment of oocytes

For nocodazole treatment, 10 mg/ml nocodazole in DMSO stock was diluted in M2 medium to give a final concentration of 20 μg/ml, and oocytes were incubated for 10 min at MI stage and collected for immunofluorescence microscopy after 9 h culture.

### Inhibition of MKlp2

After collection, oocytes were cultured in M2 medium containing 100 or 200 μM paprotrain for 12 h. Spindle phenotypes and chromosome localization were examined using a confocal microscope (Zeiss LSM 700 META, Germany).

### Rescue experiment

After cultured in M2 medium containing 200 μM paprotrain for 12 h, treated oocytes were washed five times in fresh M2 medium (2 min each wash). Oocytes were then transferred to fresh M2 medium and cultured for an additional 9 h under paraffin oil at 37°C in a 5% CO_2_ atmosphere.

### Confocal microscopy

For immunostaining, oocytes were fixed in 4% paraformaldehyde in PBS for 30 min at room temperature and then transferred to membrane permeabilization solution (0.5% Triton X-100) for 20 min. After 1 h in blocking buffer (1% BSA-supplemented PBS), oocytes were incubated overnight at 4°C or for 4 h at room temperature with a rabbit anti-MKlp2 antibody (1:25). After three washes in washing buffer (0.1% Tween 20 and 0.01% Triton X-100 in PBS), oocytes were labeled with FITC-anti-rabbit IgG or TRITC-anti-rabbit IgG (1:100) for 1 h at room temperature. For α-tubulin-FITC staining, after incubation for 1 h, oocytes were washed three times (2 min per wash) in PBS containing 0.1% Tween 20 and 0.01% Triton X-100. Samples were then co-stained with Hoechst 33342 (10 μg/ml in PBS) for 10 min, followed by three washes in washing buffer. Oocytes were mounted on glass slides and examined with a confocal laser-scanning microscope (Zeiss LSM 700 META, Germany). At least 20 oocytes were examined for each group.

### Time-lapse microscopy

After microinjection of tubulin-GFP mRNA (derived from in vitro transcription of the pRN3-b5-tubulin-GFP plasmid), oocytes were incubated in M2 medium containing Hoechst 33342 (5 ng/ml, Sigma) and paprotrain (200 μM) for imaging spindle and chromosome dynamics during oocyte maturation. Microtubule dynamics were imaged with a Perkin Elmer pre cisely Ultra VIEW VOX confocal Imaging System. Exposure times were set to a range between 200 and 800 ms, depending on the tubulin-GFP fluorescence level. The acquisition of digital time-lapse images was controlled by IP Lab (Scanalytics) or AQM6 (Andor/Kinetic-imaging) software packages. Confocal images of spindles in live oocytes were acquired with a 100× objective on a spinning disk confocal microscope (Perkin Elmer).

### Statistical analysis

At least three replicates were performed for each experiment with results expressed as means ± SEM’s. Statistical comparisons were made by independent-sample t-tests. A p-value of < 0.05 was considered significant.

## Results

### Expression and localization of MKLP2 during mouse oocyte meiotic maturation

**Figure 1:**
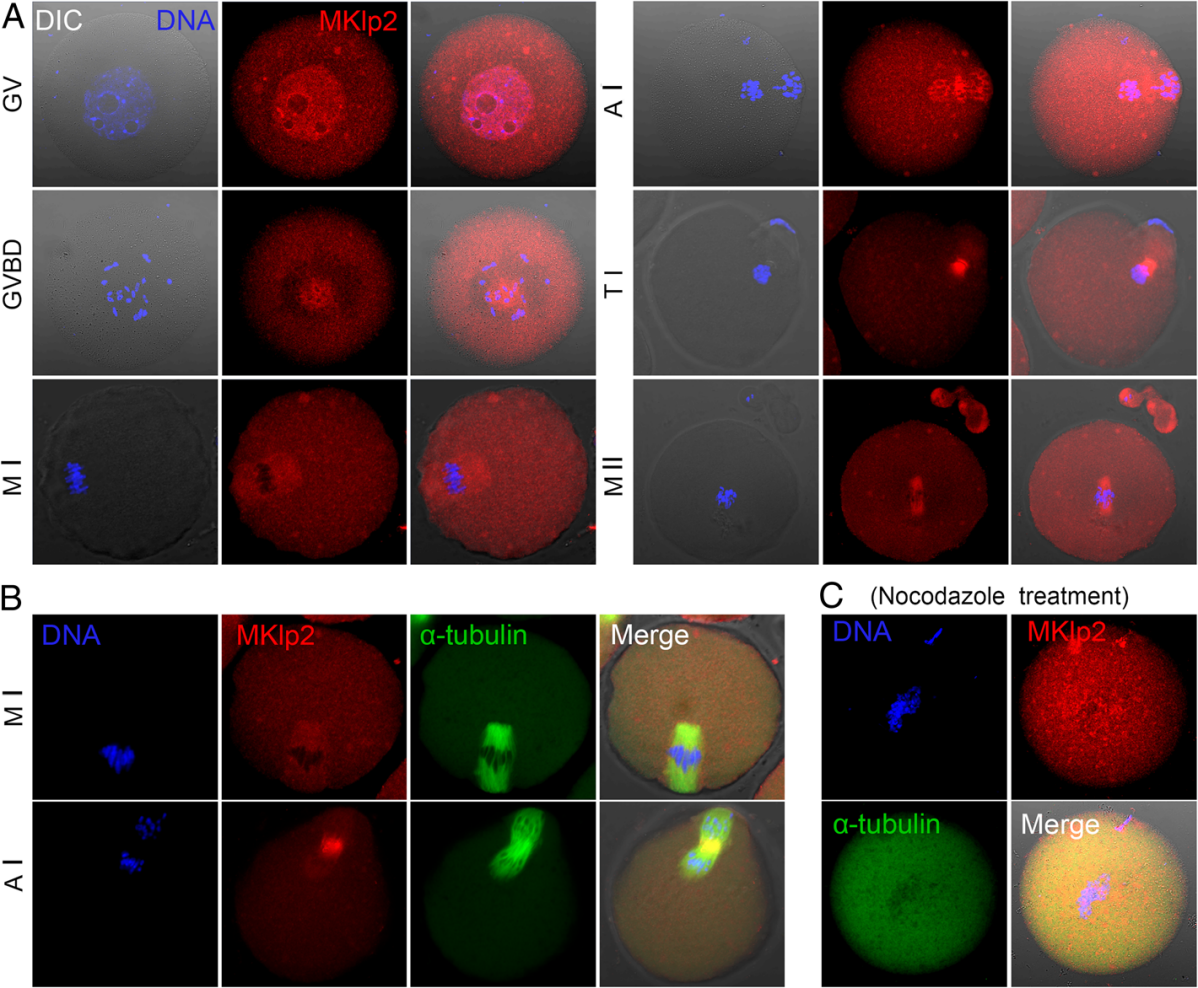
**Expression and localization of MKlp2 in mouse oocytes. (A)** Subcellular localization of MKlp2 during mouse oocyte meiotic maturation. MKlp2 antibody staining was employed to show the subcellular localization of MKlp2 in mouse oocytes. During mouse oocyte meiotic maturation, MKlp2 accumulated at the spindle and in the cytoplasm of oocytes. Red, MKlp2; blue, chromatin. **(B)** Co-localization of MKlp2 and α-tubulin. MKlp2 co-localized with spindle microtubules. Green, α-tubulin; red, MKlp2; blue, chromatin. **(C)** Localization of MKlp2 in mouse oocytes after treatment with nocodazole. Green, α-tubulin; red, MKlp2; blue, chromatin.

Oocyte samples were collected after culture for 0, 4, 8, 9.5 or 12 h, which represent the time points when most oocytes reach the GV, GVBD, MI, anaphase/telophase I (ATI) and MII stages, respectively. The subcellular localization of MKlp2 at different stages of meiotic maturation was examined using immunofluorescent staining with an MKlp2 antibody. As shown in Figure 1A, MKlp2 was localized in the germinal vesicle during the GV stage. After GVBD, MKlp2 accumulated around chromosomes and localized at spindle microtubules from the MI to the MII stage. MKlp2 signal was also detected in the cytoplasm besides spindle microtubules.

We also co-stained MKlp2 and α-tubulin at MI stage, and their signals were all overlapped, which further confirmed the localization of MKlp2 in oocytes (Figure 1B). Furthermore, we treated oocytes with the spindle-perturbing agent nocodazole at MI stage and observed a change of MKlp2 distribution. As shown in Figure 1C, after nocodazole treatment, no MKlp2 or tubulin signal could be detected, which further suggested that MKlp2 was localized to spindle microtubules.

### MKlp2 inhibition results in polar body extrusion failure

**Figure 2:**
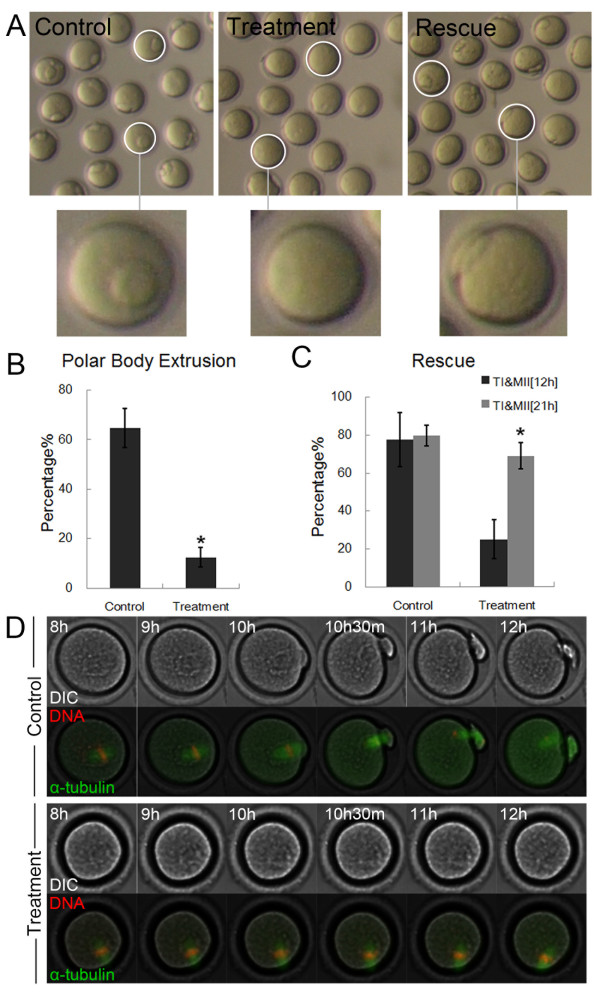
**Effects of MKlp2 disruption on polar body extrusion in mouse oocytes. (A)** Oocytes failed to extrude polar bodies after MKlp2 disruption by paprotrain treatment, whereas after rescue, oocytes developed to the MII stage. Circles in control and rescued oocytes show oocytes that reached the MII stage, whereas circles in treated oocytes show that they were arrested at an earlier stage. **(B)** Rates of polar body extrusion after MKlp2 disruption. *significantly different (p < 0.05). **(C)** Rates of oocytes remaining at the TI and MII stage after MKlp2 disruption and after rescue. *significantly different (p < 0.05). **(D)** Time-lapse microscopy of maturing oocytes after MKlp2 disruption. For controls, oocytes extruded polar bodies; whereas treated oocytes were arrested at the MI stage.

To further investigate possible roles of MKlp2 during mouse oocyte meiotic maturation, an MKlp2 inhibitor, paprotrain, was used to disrupt MKlp2 protein activity. As shown in Figure 2A, B, after being cultured for 12 h, only 12.5 ± 3.9% of treated oocytes (100 μM) (n = 102) had extruded polar bodies compared to 64.6 ± 8.0% of the control oocytes (n = 118) (p < 0.05). To demonstrate it was the disruption of MKlp2 that caused the failure of polar body extrusion, we did the rescue experiment. After an additional 9 h of culture in fresh medium, treated oocytes staying at MI and TI stage reached the MII stage (200 μM) (25.0 ± 5.5% to 69.0 ± 6.7%, n = 127), while the control group was 77.7 ± 14.3% to 79.9 ± 10.3% (n = 130) (Figure 2C).

We also employed time-lapse microscopy to observe the dynamic changes that occurred in maturing oocytes. As shown in Figure 2D, after 12 h in culture, control oocytes extruded polar bodies normally, whereas paprotrain-treated oocytes (200 μM) failed to extrude polar bodies. These results indicated that the disruption of MKlp2 caused failure of polar body extrusion.

### Cell cycle disturbances after MKlp2 disruption

**Figure 3:**
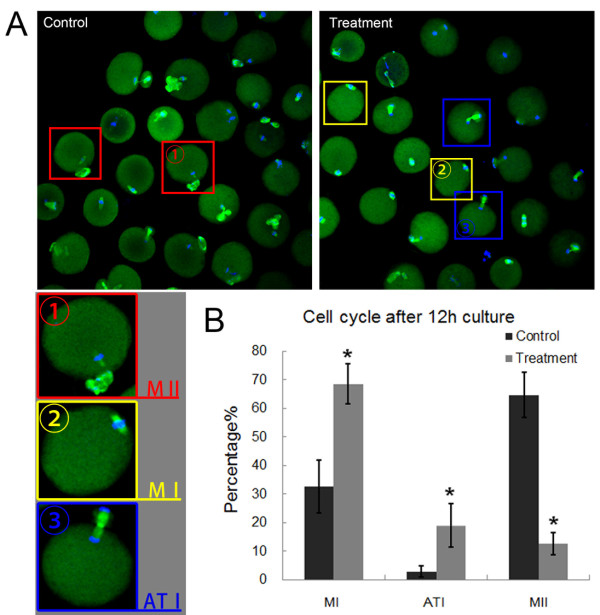
**Effects of MKlp2 disruption on cell cycle distributions during oocyte maturation. (A)** Most control oocytes reached the MII stage, whereas substantial proportions of paprotrain-treated oocytes remained in the MI and ATI stage. Red boxes in controls indicate oocytes that reached the MII stage. Yellow boxes in treated oocytes indicate that they stayed at the MI stage and blue boxes indicate oocytes arrested at the TI stage. Green, α-tubulin; blue, chromatin. **(B)** Stages after 12 h in culture for treated oocytes. Most oocytes failed to reach the MII stage and were arrested at the MI and ATI stages. *significantly different (p < 0.05).

Furthermore, we examined the proportions of oocytes that were arrested at different meiotic stages. As shown in Figure 3A, the confocal micrograph showed that most control oocytes had reached the MII stage after culture for 12 h, whereas a substantial proportion of paprotrain-treated oocytes remained in the MI and ATI stage after treated with 100 μM paprotrain for 12 h. Only 12.5 ± 3.9% of the treated oocytes (n = 102) had extruded polar bodies as compared to 64.6 ± 8.0% of the control oocytes (n = 118) (p < 0.05). Few control oocytes were arrested at the ATI stage (2.8 ± 1.9%) and MI stage (32.5 ± 9.3%), whereas a large proportion of treated oocytes remained in the MI stage (68.5 ± 6.9%) (p < 0.05) and the ATI stage (18.9 ± 7.6%) (p < 0.05) (Figure 3B). Thus, disrupting MKLp2 blocked the cell cycle progression of mouse oocytes maturation.

### MKlp2 does not affect oocyte spindle structure or chromosome alignment

**Figure 4:**
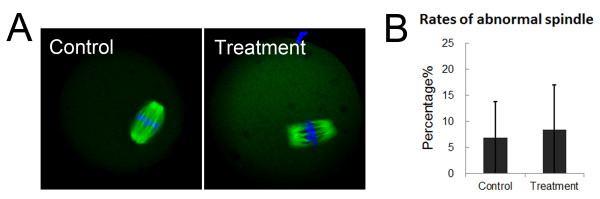
**Effects of MKlp2 disruption on oocyte MI spindle structure and chromosome alignment. (A)** Spindle structure and chromosome alignment were normal after MKlp2 disruption with paprotrain treatment. **(B)** Rate of abnormal oocytes (displaying chromosome misalignment and aberrant spindles) after MKlp2 disruption.

To explore what may have led to polar body extrusion failure, we also examined spindle structure and chromosome alignment after inhibition of MKLp2 activity. As shown by the confocal micrograph in Figure 4A, the MI spindle structure and chromosome alignment of 100 μM-paprotrain-treated oocytes were not disrupted, which was similar to what was observed with control oocytes. The proportions of abnormal oocytes (those displaying chromosome misalignments and aberrant spindles) were not significantly different between control and treated oocytes (6.9 ± 6.9%, n = 64 vs. 8.5 ± 8.5%, n = 96) (p > 0.1) (Figure 4B). Taken together, these results show that the inhibition of MKLp2 did not disrupt oocyte spindle structure and chromosome alignment.

## Discussion

In the present study, we have shown the expression and localization of MKlp2 during mouse oocyte meiotic maturation. We also demonstrated its contribution to regulating meiotic polar body extrusion.

Subito, a kinesin 6 family member and ortholog of MKlp2, participates in mitotic spindle assembly. Subito is also a protein that localizes to the central spindle during female meiosis I. It contributes to central spindle assembly and organization of the meiotic spindle in *Drosophila* oocytes [17]. Furthermore, MKlp2 has been shown to be an essential factor for cytokinesis that links Aurora B (core component of the CPC) to the equatorial cortex (or the cell cortex and the growing furrow in monopolar cytokinesis) in HeLa cells [8,18]. Meiotic polar body extrusion depends upon cytokinesis. Because the functions of MKlp2 in cytokinesis of somatic cell mitosis have been discovered, we proposed that MKlp2 was important for oocyte meiotic maturation by focusing on regulating the extrusion of first polar body.

To confirm our hypothesis, we investigated the role of MKlp2 in oocyte meiotic maturation using paprotrain, a cell-permeable acrylonitrile compound that inhibits MKlp2. We first examined the localization of MKlp2 during mouse oocyte maturation. It is possible that all members of the kinesin 6 group interact with antiparallel microtubules [19]. Our immunolocalization results were consistent with this because MKlp2 co-localized with spindle microtubules during all stages after GVBD. By using paprotrain, treated oocytes failed to extrude their first polar bodies. Most oocytes were arrested at MI or ATI stages. The higher proportion of ATI stage oocytes after treatment may reflect the roles of MKlp2 on cytokinesis. Although cytokinesis initiates, it still could not complete after MKlp2 inhibition. These results demonstrated that MKlp2 was crucial for the extrusion of first polar body, which conformed to our hypothesis.

We further explored the mechanism by which paprotrain inhibited polar body extrusion. At MI onset, the germinal vesicle envelope breaks down, chromosomes condense and microtubules reorganize progressively around them into a bipolar spindle [20]. As an important process involved in the regulation of oocyte maturation, spindle assembly and migration are absolutely necessary for the first polar body extrusion. Considering the co-localization of MKlp2 with microtubules, we hypothesized that paprotrain inhibits the oocyte maturation by disrupting the meiosis spindle assembly. Therefore, we examined spindle structure and chromosome alignment after paprotrain treatment. However, as with control oocytes, treated oocytes showed normal spindle structure and the chromosome alignment was not disrupted. Taken together, paprotrain caused failure of polar body extrusion by some mechanism other than regulating spindle assembly.

To date, the mechanism by which MKlp2 regulates oocyte maturation has not been discovered. We noticed that the MI stage arrested oocytes were also higher after MKlp2 inhibition. It has been demonstrated that Mklp2 and the CPC mutually depend on each other for midzone localization during mitosis [13]. Furthermore, after paprotrain treatment, the relocation of Aurora B and survivin (CPC component) from centromeres to the central spindle in HeLa cells is impaired [21]. Translocation of the CPC from centromeres to the spindle midzone at anaphase onset is critical for the completion of cytokinesis [20]. As an SAC component, Mad2 reportedly inhibits MKlp2 loading onto the mitotic spindle and further inhibits the ability of MKlp2 to relocate the CPC from centromeres during mitosis [16]. Because Aurora B and Mad2 are cell cycle checkpoint proteins, all evidence indicates that MKlp2 may be involved in cell cycle related processes.

Our analysis showed that cell cycle progression was disturbed, as most oocytes remained in MI stage and ATI stage after MKlp2 inhibition. Therefore, we speculate that MKlp2 regulates polar body extrusion through its effect on the cell cycle of mouse oocyte maturation. More research efforts focusing on the relationship with CPC need to be put into the underlying mechanism of MKlp2 during oocyte maturation.

## Conclusions

In summary, MKlp2 is an important microtubule-associated protein. We propose that its inhibition by a kinesin-specific MKlp2 inhibitor, paprotrain, perturbs mouse oocytes maturation by causing failure of the polar body extrusion.

## Abbreviations

CPC: Chromosomal passenger complex; MKlp2: Mitotic kinesin-like protein 2; PLK1: Polo like kinase 1; SAC: Spindle assembly checkpoint.

## Competing interests

The authors have no competing interest to declare.

## Authors’ contributions

Conceived and designed the experiments: JL SCS. Performed the experiments: JL QCW. Analyzed the data: JL SCS. Contributed reagents/materials/analysis tools: XSC ZBW NHK. Wrote the paper: JL SCS. All authors read and approved the final manuscript.
